# Persistence of Diphtheria, Hyderabad, India, 2003–2006

**DOI:** 10.3201/eid1407.071167

**Published:** 2008-07

**Authors:** Sailaja Bitragunta, Manoj V. Murhekar, Yvan J. Hutin, Padmanabha P. Penumur, Mohan D. Gupte

**Affiliations:** * National Institute of Epidemiology, Chennai, India; † Fever Hospital, Hyderabad, India

**Keywords:** Diphtheria, vaccine coverage, vaccine efficacy, India, dispatch

## Abstract

During 2003–2006, diphtheria rates in Hyderabad, India, were higher among persons 5–19 years of age, women, and Muslims than among other groups. Vaccine was efficacious among those who received >4 doses. The proportion of the population receiving boosters was low, especially among Muslims. We recommend increasing booster dose coverage.

Diphtheria is a disease caused by the exotoxin produced by *Corynebacterium diphtheriae.* The Expanded Programme of Immunization of the World Health Organization recommends 3 doses of the diphtheria, pertussis, and tetanus (DPT) vaccine starting at 6 weeks of age with additional doses of diphtheria vaccine in countries where resources permit (*1*). Many national immunization programs, including the Universal Immunization Programme of India, offer 2 booster doses at 18 months and between 54 and 72 months of age. After 3 doses of primary vaccine, protective levels of antitoxin develop in 94% to 100% of children (*1*,*2*). However, without booster doses, over time toxoid-induced antibody drops below protective levels (*2*,*3*).

In 2005, India contributed 5,826 (71%) of the 8,229 diphtheria cases reported globally (*4*). Of the total cases from India, 4,161 (71%) were from the state of Andhra Pradesh (*5*). Hyderabad, the state capital, contributed 663 (16%) of the total cases from the state (Government of Andhra Pradesh, unpub. data). The administrative coverage of primary vaccination among children 12–23 months of age (a performance indicator for Universal Immunization Programme [UIP]) ranged from 98% to 100% in the city from 1995 through 2006. We conducted a study to 1) describe the epidemiology of diphtheria in terms of time, place, and person; 2) estimate vaccine coverage; and 3) estimate diphtheria vaccine efficacy.

## The Study

Diphtheria patients identified in Hyderabad and neighboring districts are admitted to the Fever Hospital. Case-patients undergo a smear examination of the characteristic patch of thick gray membrane and samples are cultured for *C. diphtheriae*. To describe the epidemiology of diphtheria, we included cases defined as an acute febrile infection with gray-white patch in pharynx, tonsils, or fauces among residents of Hyderabad admitted to Fever Hospital during 2003–2006. We obtained data regarding age, sex, religion, month and year of occurrence, and circle (municipal administrative subdivision) of residence from the medical records of 2,685 diphtheria case-patients admitted during 2003–2006. Thirty-one case-patients died (overall case-fatality rate 1.2%). Diphtheria occurred throughout the year with lower incidences during July and August (Figure). Annual incidence increased from 11/100,000 to 23/100,000 from 2003 through 2006 (χ^2^ trend 152; p = 0.00001). Median age of case-patients was 17 years (range 9 months–80 years). Attack rates were lowest among infants, increased with age, and reached a maximum among children 10–14 years of age. Rates were higher among girls and women (Table 1). Of the 2,685 case-patients, 70% were Muslim, who had rates 3 times higher than other communities. Circle-specific attack rates ranged from 17/100,000 to 25/100,000 and were highest in the first 4 circles of the city where a predominantly Muslim population resided; this area accounted for 90% of cases in 2003–2006. During 2006, 81% of the cases were either smear or culture positive.

**Figure:**
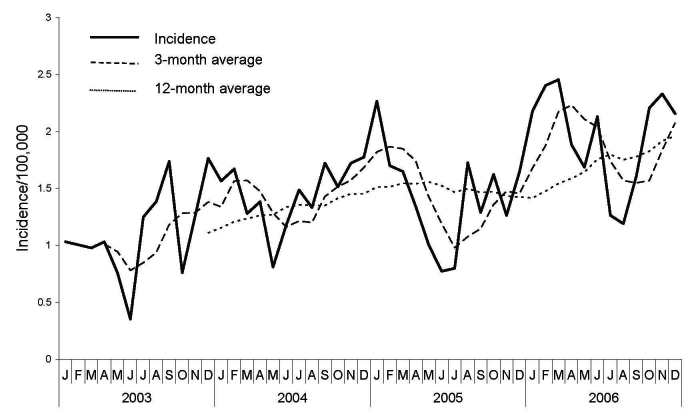
Incidence of diphtheria in Hyderabad, India, 2003–2006.

**Table 1:** Average yearly attack rate of diphtheria by age and sex, Hyderabad, India, 2003–2006

Demographic characteristics	No. cases, 2003–2006	Average population, 2003–2006	Annual attack rate/100,000 population
Age, y			
<1	8	81,050	2
2–4	116	240,450	12
5–9	455	415,673	27
10–14	530	455,426	29
15–19	431	450,408	24
20–44	1,054	1,583,569	17
>45	91	632,964	4
Sex			
Male	1,153	1,983,960	15
Female	1,532	1,875,580	20
Religion			
Non-Muslim	811	2,270,695	9
Muslim	1,874	1,588,845	29
Total	2,685	3,859,540	17

We surveyed the 7 circles of the city to estimate primary vaccination coverage among children 12–23 months of age, fourth diphtheria dose (DPT) among those 18–36 months of age, and fifth diphtheria dose (DT) among children 54–72 months of age, respectively. We selected a stratified sample of 658 children in each age group. Criteria for a completely vaccinated child were defined according to the UIP vaccination schedule by age group.

Coverage for primary vaccination, fourth, and fifth doses was 90% (95% confidence interval [CI] 89%–90%), 60% (95% CI 59%–60%), and 33% (95% CI 33%–34%), respectively. Although coverage for primary vaccination did not differ among Muslims and non-Muslims (coverage ratio 0.95, 95% CI, 0.90%–1.1%), coverage for fourth and fifth doses was lower among Muslims (coverage ratios 0.86, 95% CI 0.75%–0.99% and 0.59, 95% CI 0.5%–0.8%, respectively).

We compared laboratory-confirmed case-patients <10 years of age who lived in Hyderabad with age- and residence-matched controls. Information about educational status of parents, monthly family income, religion, and number of vaccine doses received was collected through interviews of mothers or guardians. Vaccination status was ascertained from vaccination cards or the mother’s history when a card was not available. All exposures were included in a stepwise conditional logistic regression by using Epi Info (Centers for Disease Control and Prevention, Atlanta, GA, USA). Vaccine efficacy (%) was calculated by using the 1 – odds ratio formula (*6*). We included all children in the analysis to estimate vaccine efficacy for the first 4 doses. However, to estimate the efficacy of 5 doses of diphtheria vaccine, we restricted the analysis to children 5–10 years of age because the fifth dose of the vaccine is given to children >4.5 years of age.

We included 123 case-patients in the case-control study. Only 20 (16%) controls and 23 (19%) case-patients had a vaccination card. The median age of case-patients was 7 years and 50% were girls. Twenty-one children (17%) were younger than 5 years of age. When adjusted for religion, family income, and literacy status of parents, vaccine efficacy increased from 49% (95% CI 0%–80%) for 3 doses to 65% (95% CI 8%–87%) for 4 doses. Among children >5 years of age, efficacy for 5 doses was 91% (95% CI 68%–98%) (Table 2).

**Table 2:** Number of doses of diphtheria vaccine received by diphtheria case-patients and matched controls, Hyderabad, India, 2006*

No. doses received	Case-patients				Controls			Odds ratio estimate	Vaccine efficacy estimate, % (95% CI)
	<5 y of age	>5 y of age	Total		<5 y of age	>5 y of age	Total		
0	6	20	26		2	11	13	Reference	Reference
1	0	7	7		1	2	3	1.4 (0.22–8.9)	0 (0–78)
2	2	6	8		0	2	2	2.1 (0.37–12)	0 (0–63)
3	6	34	40		8	26	34	0.51 (0.20–1.3)	49 (0–80)
4†	7	27	34		10	30	40	0.35 (0.13–92)	65 (8–87)
5‡	NA	8	8		NA	31	31	0.09 (0.02–0.33)	91§ (68–98)

*Conditional logistic regression taking into account vaccine doses, religion, family income, and parental literacy. CI, confidence interval; NA, not applicable. †Booster at 18 mo of age. ‡Booster at 5 y of age. §Vaccine efficacy among children >5 y of age; younger children could not have received that booster.

## Conclusions

Our results indicate that in Hyderabad, diphtheria mainly affected children 5–19 years of age, girls and women, and the Muslim population. Receiving a fourth and fifth doses of the vaccine was needed for protection against the disease. Coverage of primary vaccination was adequate in the city whereas, coverage for the boosters was low.

Low booster coverage, especially among Muslims, might have influenced herd immunity and thereby contributed to higher attack rates among this community. This factor was likely an important reason for persistence of diphtheria in Hyderabad. Similar phenomena were observed in countries where diphtheria reemerged after successful control with vaccination (*2*,*7*). Several studies have reported vaccine efficacies ranging from 95% to 98% for 3 doses and from 90% to 99.9% for 5 doses (*8*,*9*). Two factors may explain the lower efficacy observed in our study. First, misclassification may have occurred when assessing vaccination status of children that mainly relied on a mother’s recollection of the child’s history. Second, program quality issues in vaccine supply or in cold-chain maintenance may have affected efficacy in Hyderabad. However, an evaluation of the universal immunization program conducted in 2006 in Hyderabad did not identify any gaps in cold-chain maintenance in the public health sector (Government of Andhra Pradesh, unpub. data). Thus, we concluded that misclassification with respect to assessment of vaccination status (using only the mother’s recollection) probably explains the low vaccine efficacy that we observed.

Two factors could also explain the lower booster coverage among Muslims: 1) issues concerning the offer of vaccine by the health services or 2) issues concerning vaccine demand. However, primary vaccination coverage was identical among Muslims and non-Muslims in Hyderabad. This suggests that, initially, the demand for primary vaccination was identical in all communities, but that the health system was not able to retain the same demand for boosters in the Muslim community.

Our study had 2 main limitations. First, we only included patients admitted to Fever Hospital. Patients with milder symptoms who might not have sought treatment at the hospital were not considered. This situation may have led to an underestimation of attack rates but would not have led to different conclusions about the persistence of the disease. Second, a large proportion of children did not have vaccination cards, which may also have affected our vaccine efficacy estimates. We tried to address this factor by comparing vaccination status with the child’s developmental milestones**.**

On the basis of our study results, we propose recommendations for control of diphtheria in Hyderabad. First, coverage for boosters must be improved, with special emphasis on the 4 administrative areas with high attack rates. Such efforts should be conducted among the Muslim community in particular. Second, mothers must be made more aware of the importance of booster doses. Again, these efforts should focus on the Muslim community. Third, because attack rates were high among adolescents, tetanus toxoid (administered to school children at 10–15 years of age) could be replaced with a combined tetanus-diphtheria vaccine. Fourth, coverage of boosters could be considered as performance indicators to improve the immunization program.
